# 4-(4-Octyloxybenzo­yloxy)benzoic acid

**DOI:** 10.1107/S1600536809003298

**Published:** 2009-01-31

**Authors:** Khushi Muhammad, M. Khawar Rauf, Masahiro Ebihara, Shahid Hameed

**Affiliations:** a Department of Chemistry, Quaid-i-Azam University, Islamabad 45320, Pakistan; b Department of Chemistry, Faculty of Engineering, Gifu University, Yanagido, Gifu 501-1193, Japan

## Abstract

The title compound, C_22_H_26_O_5_, is an important inter­mediate for the synthesis of side-chain ligands for polymeric liquid crystals. The octyl group is coplanar with the central C_6_O moiety, where the maximum deviation of a C atom in the octyl group from the C_6_O plane is 0.161 (5) Å. The crystal structure is stabilized by inter­molecular O—H⋯O hydrogen bonds.

## Related literature

For studies of aromatic carboxylic acids and their applications, see: Naoum *et al.* (2008 ▶); Nazir *et al.* (2008*a* ▶,*b* ▶); Gabert *et al.* (2006 ▶); Aranzazu *et al.* (2006 ▶); Hussain *et al.* (2005 ▶); Shafiq *et al.* (2005 ▶); Ahmad *et al.* (2003 ▶); Ribeiro *et al.* (2008 ▶); Hameed & Rama (2004 ▶); For related structures, see: Muhammad *et al.* (2008 ▶); Hartung *et al.* (1997 ▶)
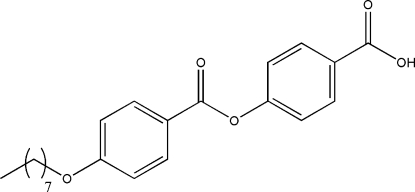


## Experimental

### 

#### Crystal data

C_22_H_26_O_5_
*M* *_r_* = 370.43
Monoclinic, 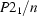

*a* = 13.528 (8) Å
*b* = 7.245 (4) Å
*c* = 20.903 (12) Å
β = 111.407 (8)°
*V* = 1907.5 (18) Å^3^
*Z* = 4
Mo *K*α radiation
μ = 0.09 mm^−1^
*T* = 123 K
0.40 × 0.30 × 0.15 mm

#### Data collection

Rigaku/MSC Mercury CCD diffractometer
Absorption correction: none
14669 measured reflections
4358 independent reflections
3870 reflections with *I* > 2σ(*I*)
*R*_int_ = 0.048

#### Refinement

*R*[*F* ^2^ > 2σ(*F* ^2^)] = 0.081
*wR*(*F* ^2^) = 0.138
*S* = 1.31
4358 reflections
247 parameters
H-atom parameters constrained
Δρ_max_ = 0.28 e Å^−3^
Δρ_min_ = −0.19 e Å^−3^

### 

Data collection: *CrystalClear* (Molecular Structure Corporation & Rigaku, 2001 ▶); cell refinement: *CrystalClear*; data reduction: *TEXSAN* (Molecular Structure Corporation & Rigaku, 2004 ▶); program(s) used to solve structure: *SIR97* (Altomare *et al.*, 1999 ▶); program(s) used to refine structure: *SHELXL97* (Sheldrick, 2008 ▶); molecular graphics: *ORTEPII* (Johnson, 1976 ▶); software used to prepare material for publication: *SHELXL97* and *TEXSAN*.

## Supplementary Material

Crystal structure: contains datablocks I, global. DOI: 10.1107/S1600536809003298/hg2473sup1.cif

Structure factors: contains datablocks I. DOI: 10.1107/S1600536809003298/hg2473Isup2.hkl

Additional supplementary materials:  crystallographic information; 3D view; checkCIF report

## Figures and Tables

**Table 1:** Hydrogen-bond geometry (Å, °)

*D*—H⋯*A*	*D*—H	H⋯*A*	*D*⋯*A*	*D*—H⋯*A*
O3—H3O⋯O4^i^	0.84	1.85	2.659 (3)	161
O4—H4O⋯O3^i^	0.84	1.83	2.659 (3)	171

Symmetry code: (i) 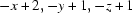.

**Fig. 1.**
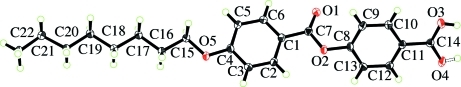
Molecular structure of (I) showing atom-labelling scheme and displacement ellipsoids at the 30% probability level.

**Fig. 2.**
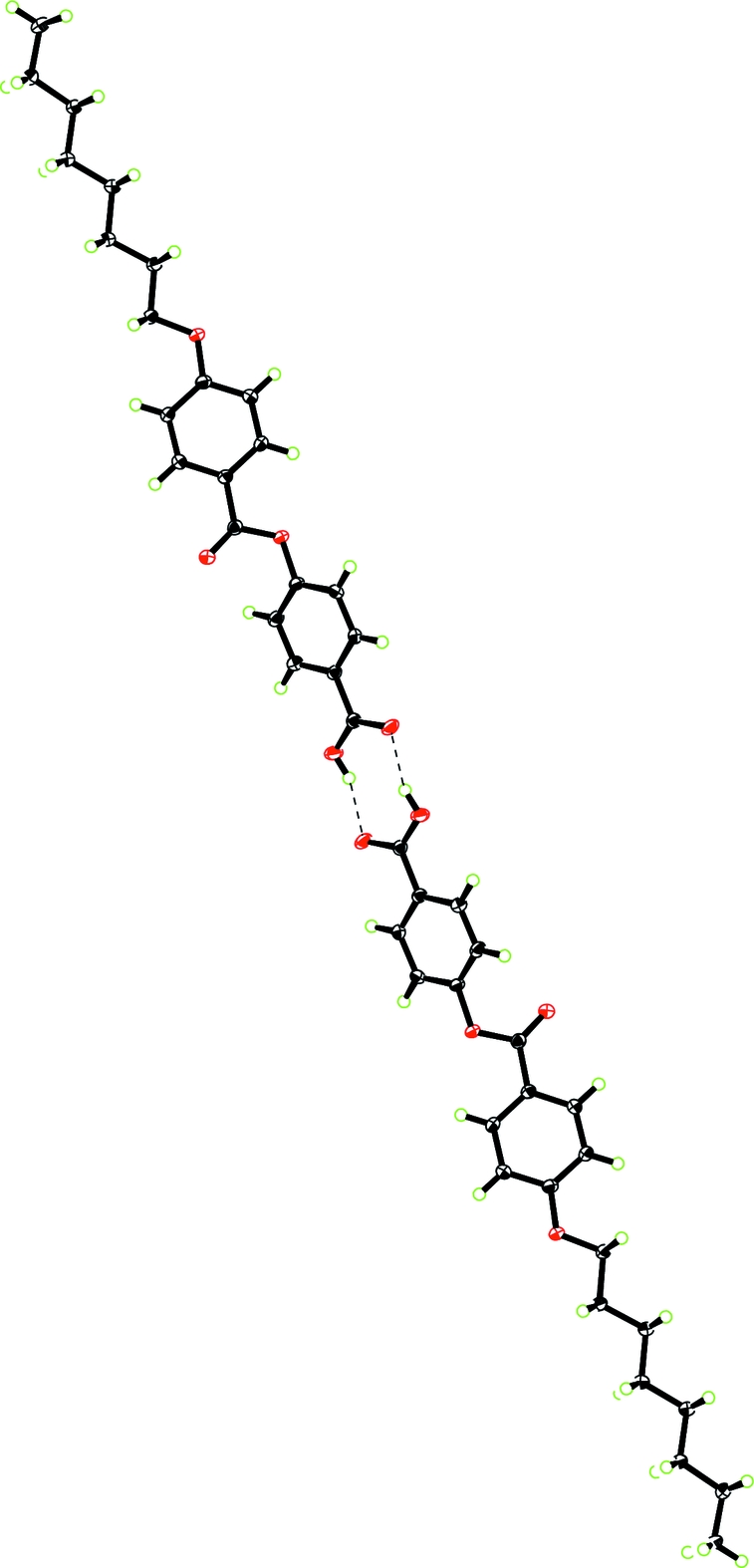
Showing hydrogen bonded molecules through O—H···O.

## supplementary crystallographic information

### Comment

Aromatic carboxylic acids bearing different substituents have been investigated for their liquid crystalline properties (Naoum *et al.*, 2008; Nazir *et al.*, 2008*a*,*b*). Such acids have been utilized in the synthesis of both side-chain (Gabert *et al.*, 2006) and main-chain (Aranzazu *et al.*, 2006) liquid crystal polymers. In addition to their use as intermediates in the synthesis of a large number of organic compounds (Hussain *et al.*, 2005; Shafiq *et al.*, 2005; Ahmad *et al.*, 2003), carboxylic acids has also been used in the pharmaceutical industry (Ribeiro *et al.*, 2008; Hameed & Rama 2004). The title compound (I) was synthesized as an intermediate in the synthesis of side-chain liquid crystal polymers. In the present report, the crystal structure of (I) is being presented. Bond lengths and angles are within the normal ranges as given for benzoyloxybenzoic acids (Muhammad *et al.*, 2008; Hartung *et al.*, 1997). The C7—O1 and C7—O2 bond lengths are 1.205 (3) and 1.369 (3) Å, respectively, that reflect their double and single bond character. The very similar bond lengths of C14—O4 and C14—O3, 1.287 (3) and 1.261 (3) Å, are due to disorder of CO_2_H moiety. The octyl group is coplanar with the central C_6_O moiety where the *max* dviation of C atom in octyl group from the C_6_O moiety is 0.161 (5) Å. Two molecules related by an inversion center form a dimer *via* two hydrogen bonds composed of two carboxyl groups as shown in Fig. 2.

### Experimental

To a solution of 4-hydroxybenzaldehyde (0.032 moles) in 50 ml of triethylamine (TEA), was added an equivalent amount of 4-octyloxybenzoylchloride with stirring and the mixture heated at 60°C for 1 h. The excess TEA was removed *in vacuo* and the product, after recrystallization from hot ethanol, was subjected to KMnO_4_ oxidation.The 4-(4-octyloxybenzoyloxy)benzaldehyde (0.025 moles) was dissolved in acetone (100 ml) and aqueous KMnO_4_ (0.025 moles) was added dropwise at room temperature with stirring. The stirring was continued for three hours when the reaction mixture was filtered and the filtrate acidified using 6*M* HCl. The precipitated product was purified by recrystallization from acetone.

### Refinement

The O-bound H atom was refined isotropically. All the other H atoms were placed in idealized positions and treated as riding atoms, with C—H distance in the range 0.95–0.99 Å and *U*_iso_(H) = 1.2*U*_eq_(C) or 1.5*U*_eq_(C).

### Crystal data

**Table:**

C_22_H_26_O_5_	*F*(000) = 792
*M**_r_* = 370.43	*D*_x_ = 1.290 Mg m^−^^3^
Monoclinic, *P*2_1_/*n*	Mo *K*α radiation, λ = 0.71070 Å
Hall symbol: -P 2yn	Cell parameters from 4339 reflections
*a* = 13.528 (8) Å	θ = 3.0–27.5°
*b* = 7.245 (4) Å	µ = 0.09 mm^−^^1^
*c* = 20.903 (12) Å	*T* = 123 K
β = 111.407 (8)°	Chip, colourless
*V* = 1907.5 (18) Å^3^	0.40 × 0.30 × 0.15 mm
*Z* = 4	

### Data collection

**Table:**

Rigaku/MSC Mercury CCD diffractometer	3870 reflections with *I* > 2σ(*I*)
Radiation source: fine-focus sealed tube	*R*_int_ = 0.048
graphite	θ_max_ = 27.5°, θ_min_ = 3.2°
Detector resolution: 14.62 pixels mm^-1^	*h* = −17→15
ω scans	*k* = −7→9
14669 measured reflections	*l* = −25→27
4358 independent reflections	

### Refinement

**Table:**

Refinement on *F*^2^	Primary atom site location: structure-invariant direct methods
Least-squares matrix: full	Secondary atom site location: difference Fourier map
*R*[*F*^2^ > 2σ(*F*^2^)] = 0.081	Hydrogen site location: inferred from neighbouring sites
*wR*(*F*^2^) = 0.138	H-atom parameters constrained
*S* = 1.31	*w* = 1/[σ^2^(*F*_o_^2^) + (0.0263*P*)^2^ + 1.5055*P*] where *P* = (*F*_o_^2^ + 2*F*_c_^2^)/3
4358 reflections	(Δ/σ)_max_ < 0.001
247 parameters	Δρ_max_ = 0.28 e Å^−^^3^
0 restraints	Δρ_min_ = −0.19 e Å^−^^3^

### Special details

**Table:**

Geometry. All e.s.d.'s (except the e.s.d. in the dihedral angle between two l.s. planes) are estimated using the full covariance matrix. The cell e.s.d.'s are taken into account individually in the estimation of e.s.d.'s in distances, angles and torsion angles; correlations between e.s.d.'s in cell parameters are only used when they are defined by crystal symmetry. An approximate (isotropic) treatment of cell e.s.d.'s is used for estimating e.s.d.'s involving l.s. planes.
Refinement. Refinement of *F*^2^ against ALL reflections. The weighted *R*-factor *wR* and goodness of fit *S* are based on *F*^2^, conventional *R*-factors *R* are based on *F*, with *F* set to zero for negative *F*^2^. The threshold expression of *F*^2^ > σ(*F*^2^) is used only for calculating *R*-factors(gt) *etc*. and is not relevant to the choice of reflections for refinement. *R*-factors based on *F*^2^ are statistically about twice as large as those based on *F*, and *R*- factors based on ALL data will be even larger.

### Fractional atomic coordinates and isotropic or equivalent isotropic displacement parameters (Å^2^)

**Table:**

	*x*	*y*	*z*	*U*_iso_*/*U*_eq_	Occ. (<1)
C1	0.22298 (16)	0.5733 (3)	0.30577 (10)	0.0181 (4)	
C2	0.19154 (16)	0.5148 (3)	0.23723 (10)	0.0218 (5)	
H2	0.2435	0.4795	0.2189	0.026*	
C3	0.08502 (17)	0.5086 (3)	0.19644 (11)	0.0239 (5)	
H3	0.0638	0.4695	0.1500	0.029*	
C4	0.00858 (16)	0.5596 (3)	0.22337 (10)	0.0202 (5)	
C5	0.03873 (16)	0.6186 (3)	0.29139 (10)	0.0204 (5)	
H5	−0.0132	0.6530	0.3099	0.024*	
C6	0.14626 (17)	0.6261 (3)	0.33158 (10)	0.0211 (5)	
H6	0.1676	0.6683	0.3777	0.025*	
C7	0.33624 (16)	0.5860 (3)	0.35119 (10)	0.0189 (4)	
O1	0.36934 (12)	0.6558 (2)	0.40736 (7)	0.0245 (4)	
O2	0.40082 (11)	0.5073 (2)	0.32137 (7)	0.0231 (4)	
C8	0.51107 (16)	0.5074 (3)	0.35729 (10)	0.0198 (5)	
C9	0.55644 (17)	0.4315 (3)	0.42229 (11)	0.0218 (5)	
H9	0.5133	0.3826	0.4453	0.026*	
C10	0.66633 (16)	0.4284 (3)	0.45304 (11)	0.0201 (4)	
H10	0.6987	0.3783	0.4979	0.024*	
C11	0.72990 (16)	0.4980 (3)	0.41892 (10)	0.0174 (4)	
C12	0.68191 (16)	0.5711 (3)	0.35287 (10)	0.0191 (4)	
H12	0.7246	0.6174	0.3291	0.023*	
C13	0.57211 (17)	0.5762 (3)	0.32203 (10)	0.0210 (5)	
H13	0.5392	0.6262	0.2772	0.025*	
C14	0.84738 (16)	0.4969 (3)	0.45311 (10)	0.0182 (4)	
O3	0.88859 (12)	0.4343 (2)	0.51339 (7)	0.0257 (4)	
H3O	0.9550	0.4364	0.5257	0.038*	0.50
O4	0.90241 (12)	0.5614 (2)	0.41929 (8)	0.0258 (4)	
H4O	0.9671	0.5599	0.4443	0.039*	0.50
O5	−0.09388 (11)	0.5459 (2)	0.17868 (7)	0.0249 (4)	
C15	−0.17837 (16)	0.5852 (3)	0.20264 (10)	0.0205 (5)	
H15A	−0.1738	0.7146	0.2188	0.025*	
H15B	−0.1743	0.5023	0.2412	0.025*	
C16	−0.28096 (16)	0.5541 (3)	0.14232 (10)	0.0196 (4)	
H16A	−0.2839	0.4239	0.1273	0.024*	
H16B	−0.2814	0.6332	0.1036	0.024*	
C17	−0.37919 (16)	0.5958 (3)	0.15880 (10)	0.0214 (5)	
H17A	−0.3764	0.7258	0.1740	0.026*	
H17B	−0.3792	0.5161	0.1973	0.026*	
C18	−0.48182 (16)	0.5646 (3)	0.09742 (10)	0.0212 (5)	
H18A	−0.4790	0.6383	0.0582	0.025*	
H18B	−0.4857	0.4329	0.0841	0.025*	
C19	−0.58294 (16)	0.6147 (3)	0.10966 (10)	0.0219 (5)	
H19A	−0.5865	0.5417	0.1488	0.026*	
H19B	−0.5806	0.7470	0.1221	0.026*	
C20	−0.68233 (16)	0.5782 (3)	0.04655 (10)	0.0205 (5)	
H20A	−0.6752	0.6432	0.0068	0.025*	
H20B	−0.6864	0.4443	0.0364	0.025*	
C21	−0.78576 (17)	0.6380 (4)	0.05333 (11)	0.0264 (5)	
H21A	−0.7825	0.7718	0.0635	0.032*	
H21B	−0.7944	0.5718	0.0924	0.032*	
C22	−0.88171 (17)	0.5991 (3)	−0.01178 (12)	0.0261 (5)	
H22A	−0.8708	0.6557	−0.0513	0.039*	
H22B	−0.9456	0.6510	−0.0070	0.039*	
H22C	−0.8904	0.4654	−0.0189	0.039*	

### Atomic displacement parameters (Å^2^)

**Table:**

	*U*^11^	*U*^22^	*U*^33^	*U*^12^	*U*^13^	*U*^23^
C1	0.0150 (10)	0.0197 (11)	0.0193 (9)	−0.0018 (8)	0.0060 (8)	−0.0005 (8)
C2	0.0153 (10)	0.0294 (13)	0.0218 (10)	0.0000 (9)	0.0080 (8)	−0.0036 (9)
C3	0.0195 (11)	0.0332 (13)	0.0178 (10)	−0.0005 (10)	0.0055 (8)	−0.0049 (9)
C4	0.0153 (10)	0.0229 (12)	0.0202 (10)	−0.0004 (9)	0.0039 (8)	−0.0010 (9)
C5	0.0156 (10)	0.0266 (12)	0.0201 (10)	0.0003 (9)	0.0078 (8)	0.0002 (8)
C6	0.0190 (11)	0.0273 (12)	0.0170 (10)	0.0002 (9)	0.0066 (8)	−0.0008 (8)
C7	0.0176 (10)	0.0195 (11)	0.0204 (10)	−0.0012 (9)	0.0080 (8)	0.0012 (8)
O1	0.0180 (8)	0.0345 (10)	0.0195 (7)	−0.0007 (7)	0.0049 (6)	−0.0042 (7)
O2	0.0122 (7)	0.0339 (10)	0.0212 (7)	0.0011 (7)	0.0037 (6)	−0.0064 (7)
C8	0.0124 (10)	0.0240 (12)	0.0208 (10)	0.0011 (9)	0.0035 (8)	−0.0050 (9)
C9	0.0169 (10)	0.0244 (12)	0.0255 (10)	−0.0020 (9)	0.0095 (8)	−0.0002 (9)
C10	0.0174 (10)	0.0210 (11)	0.0210 (10)	0.0011 (9)	0.0059 (8)	0.0006 (9)
C11	0.0153 (10)	0.0159 (11)	0.0206 (9)	0.0005 (8)	0.0060 (8)	−0.0026 (8)
C12	0.0184 (10)	0.0219 (11)	0.0179 (9)	0.0005 (9)	0.0077 (8)	−0.0030 (8)
C13	0.0191 (10)	0.0266 (12)	0.0151 (9)	0.0021 (9)	0.0036 (8)	−0.0026 (8)
C14	0.0180 (10)	0.0172 (10)	0.0200 (9)	0.0004 (9)	0.0075 (8)	−0.0022 (8)
O3	0.0160 (7)	0.0338 (10)	0.0232 (8)	0.0005 (7)	0.0024 (6)	0.0036 (7)
O4	0.0140 (7)	0.0347 (10)	0.0291 (8)	−0.0015 (7)	0.0084 (6)	0.0029 (7)
O5	0.0130 (7)	0.0395 (10)	0.0206 (7)	0.0009 (7)	0.0042 (6)	−0.0046 (7)
C15	0.0158 (10)	0.0259 (12)	0.0203 (10)	0.0000 (9)	0.0071 (8)	0.0012 (9)
C16	0.0154 (10)	0.0211 (11)	0.0205 (10)	0.0005 (9)	0.0045 (8)	−0.0001 (8)
C17	0.0162 (10)	0.0266 (12)	0.0194 (10)	0.0008 (9)	0.0042 (8)	−0.0015 (9)
C18	0.0170 (10)	0.0253 (12)	0.0189 (10)	−0.0003 (9)	0.0038 (8)	−0.0007 (9)
C19	0.0173 (10)	0.0267 (13)	0.0201 (10)	−0.0004 (9)	0.0050 (8)	−0.0019 (9)
C20	0.0158 (10)	0.0248 (12)	0.0198 (10)	−0.0002 (9)	0.0050 (8)	0.0003 (9)
C21	0.0191 (11)	0.0376 (14)	0.0231 (11)	0.0044 (10)	0.0084 (9)	0.0004 (10)
C22	0.0152 (10)	0.0282 (13)	0.0333 (12)	0.0005 (9)	0.0068 (9)	−0.0001 (10)

### Geometric parameters (Å, °)

**Table:**

C1—C6	1.387 (3)	O3—H3O	0.8400
C1—C2	1.403 (3)	O4—H4O	0.8400
C1—C7	1.482 (3)	O5—C15	1.434 (3)
C2—C3	1.382 (3)	C15—C16	1.513 (3)
C2—H2	0.9500	C15—H15A	0.9900
C3—C4	1.396 (3)	C15—H15B	0.9900
C3—H3	0.9500	C16—C17	1.520 (3)
C4—O5	1.363 (2)	C16—H16A	0.9900
C4—C5	1.396 (3)	C16—H16B	0.9900
C5—C6	1.390 (3)	C17—C18	1.525 (3)
C5—H5	0.9500	C17—H17A	0.9900
C6—H6	0.9500	C17—H17B	0.9900
C7—O1	1.205 (3)	C18—C19	1.525 (3)
C7—O2	1.369 (3)	C18—H18A	0.9900
O2—C8	1.404 (2)	C18—H18B	0.9900
C8—C9	1.384 (3)	C19—C20	1.524 (3)
C8—C13	1.385 (3)	C19—H19A	0.9900
C9—C10	1.388 (3)	C19—H19B	0.9900
C9—H9	0.9500	C20—C21	1.520 (3)
C10—C11	1.397 (3)	C20—H20A	0.9900
C10—H10	0.9500	C20—H20B	0.9900
C11—C12	1.398 (3)	C21—C22	1.526 (3)
C11—C14	1.486 (3)	C21—H21A	0.9900
C12—C13	1.387 (3)	C21—H21B	0.9900
C12—H12	0.9500	C22—H22A	0.9800
C13—H13	0.9500	C22—H22B	0.9800
C14—O3	1.261 (3)	C22—H22C	0.9800
C14—O4	1.287 (3)		
			
C6—C1—C2	119.32 (19)	O5—C15—H15A	110.4
C6—C1—C7	118.64 (19)	C16—C15—H15A	110.4
C2—C1—C7	122.02 (19)	O5—C15—H15B	110.4
C3—C2—C1	119.9 (2)	C16—C15—H15B	110.4
C3—C2—H2	120.0	H15A—C15—H15B	108.6
C1—C2—H2	120.0	C15—C16—C17	113.15 (18)
C2—C3—C4	120.1 (2)	C15—C16—H16A	108.9
C2—C3—H3	119.9	C17—C16—H16A	108.9
C4—C3—H3	119.9	C15—C16—H16B	108.9
O5—C4—C5	124.50 (19)	C17—C16—H16B	108.9
O5—C4—C3	114.96 (19)	H16A—C16—H16B	107.8
C5—C4—C3	120.54 (19)	C16—C17—C18	112.50 (18)
C6—C5—C4	118.7 (2)	C16—C17—H17A	109.1
C6—C5—H5	120.7	C18—C17—H17A	109.1
C4—C5—H5	120.7	C16—C17—H17B	109.1
C1—C6—C5	121.39 (19)	C18—C17—H17B	109.1
C1—C6—H6	119.3	H17A—C17—H17B	107.8
C5—C6—H6	119.3	C19—C18—C17	114.88 (18)
O1—C7—O2	123.14 (19)	C19—C18—H18A	108.5
O1—C7—C1	125.5 (2)	C17—C18—H18A	108.5
O2—C7—C1	111.40 (17)	C19—C18—H18B	108.5
C7—O2—C8	119.08 (16)	C17—C18—H18B	108.5
C9—C8—C13	121.90 (19)	H18A—C18—H18B	107.5
C9—C8—O2	121.9 (2)	C20—C19—C18	112.06 (18)
C13—C8—O2	116.03 (18)	C20—C19—H19A	109.2
C8—C9—C10	118.5 (2)	C18—C19—H19A	109.2
C8—C9—H9	120.8	C20—C19—H19B	109.2
C10—C9—H9	120.8	C18—C19—H19B	109.2
C9—C10—C11	120.9 (2)	H19A—C19—H19B	107.9
C9—C10—H10	119.6	C21—C20—C19	114.91 (18)
C11—C10—H10	119.6	C21—C20—H20A	108.5
C10—C11—C12	119.38 (19)	C19—C20—H20A	108.5
C10—C11—C14	120.15 (19)	C21—C20—H20B	108.5
C12—C11—C14	120.46 (19)	C19—C20—H20B	108.5
C13—C12—C11	120.1 (2)	H20A—C20—H20B	107.5
C13—C12—H12	119.9	C20—C21—C22	112.13 (19)
C11—C12—H12	119.9	C20—C21—H21A	109.2
C8—C13—C12	119.24 (19)	C22—C21—H21A	109.2
C8—C13—H13	120.4	C20—C21—H21B	109.2
C12—C13—H13	120.4	C22—C21—H21B	109.2
O3—C14—O4	123.08 (19)	H21A—C21—H21B	107.9
O3—C14—C11	119.19 (19)	C21—C22—H22A	109.5
O4—C14—C11	117.73 (18)	C21—C22—H22B	109.5
C14—O3—H3O	109.5	H22A—C22—H22B	109.5
C14—O4—H4O	109.5	C21—C22—H22C	109.5
C4—O5—C15	119.13 (17)	H22A—C22—H22C	109.5
O5—C15—C16	106.55 (17)	H22B—C22—H22C	109.5
			
C6—C1—C2—C3	−0.6 (3)	C9—C10—C11—C12	−0.2 (3)
C7—C1—C2—C3	−178.8 (2)	C9—C10—C11—C14	178.9 (2)
C1—C2—C3—C4	−0.3 (4)	C10—C11—C12—C13	0.8 (3)
C2—C3—C4—O5	−179.2 (2)	C14—C11—C12—C13	−178.3 (2)
C2—C3—C4—C5	0.5 (4)	C9—C8—C13—C12	−0.8 (3)
O5—C4—C5—C6	179.9 (2)	O2—C8—C13—C12	−176.44 (19)
C3—C4—C5—C6	0.2 (3)	C11—C12—C13—C8	−0.3 (3)
C2—C1—C6—C5	1.4 (3)	C10—C11—C14—O3	−0.8 (3)
C7—C1—C6—C5	179.6 (2)	C12—C11—C14—O3	178.3 (2)
C4—C5—C6—C1	−1.2 (3)	C10—C11—C14—O4	179.5 (2)
C6—C1—C7—O1	−7.8 (4)	C12—C11—C14—O4	−1.4 (3)
C2—C1—C7—O1	170.4 (2)	C5—C4—O5—C15	−3.2 (3)
C6—C1—C7—O2	172.24 (19)	C3—C4—O5—C15	176.5 (2)
C2—C1—C7—O2	−9.6 (3)	C4—O5—C15—C16	−178.78 (19)
O1—C7—O2—C8	−0.1 (3)	O5—C15—C16—C17	−178.28 (18)
C1—C7—O2—C8	179.84 (18)	C15—C16—C17—C18	179.72 (19)
C7—O2—C8—C9	56.4 (3)	C16—C17—C18—C19	−176.7 (2)
C7—O2—C8—C13	−128.0 (2)	C17—C18—C19—C20	−179.4 (2)
C13—C8—C9—C10	1.4 (3)	C18—C19—C20—C21	−175.8 (2)
O2—C8—C9—C10	176.7 (2)	C19—C20—C21—C22	179.5 (2)
C8—C9—C10—C11	−0.8 (3)		

### Hydrogen-bond geometry (Å, °)

**Table:**

*D*—H···*A*	*D*—H	H···*A*	*D*···*A*	*D*—H···*A*
O3—H3O···O4^i^	0.84	1.85	2.659 (3)	161
O4—H4O···O3^i^	0.84	1.83	2.659 (3)	171

Symmetry codes: (i) −*x*+2, −*y*+1, −*z*+1.
